# Supplementary material for: Spatial Metagenomic Analysis in Understanding the Microbial Diversity of Thar Desert
Source: Biology (Basel). 2022 Mar 17;11(3):461. doi: 10.3390/biology11030461 (PMC8945486; doi:10.3390/biology11030461)
Supplement: Supplementary file 1 [file biology-11-00461-s001.zip › Supplementary Figures.pdf]

Supplementary Figure Files

Figure S1. Histogram representing contig length distribution

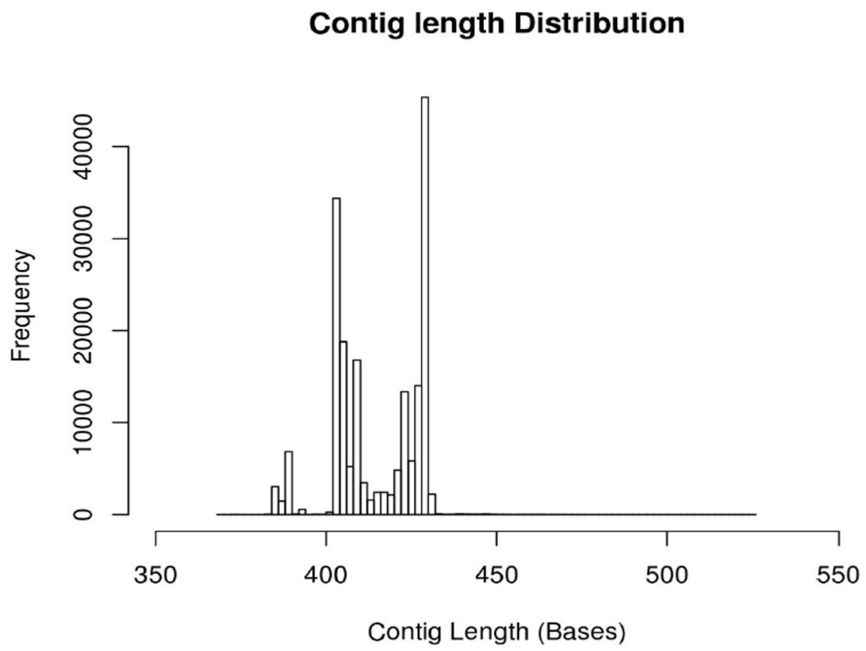

Figure S2. Rarefaction curve shows the measure of diversity that has been captured by a given number of reads in a six sample

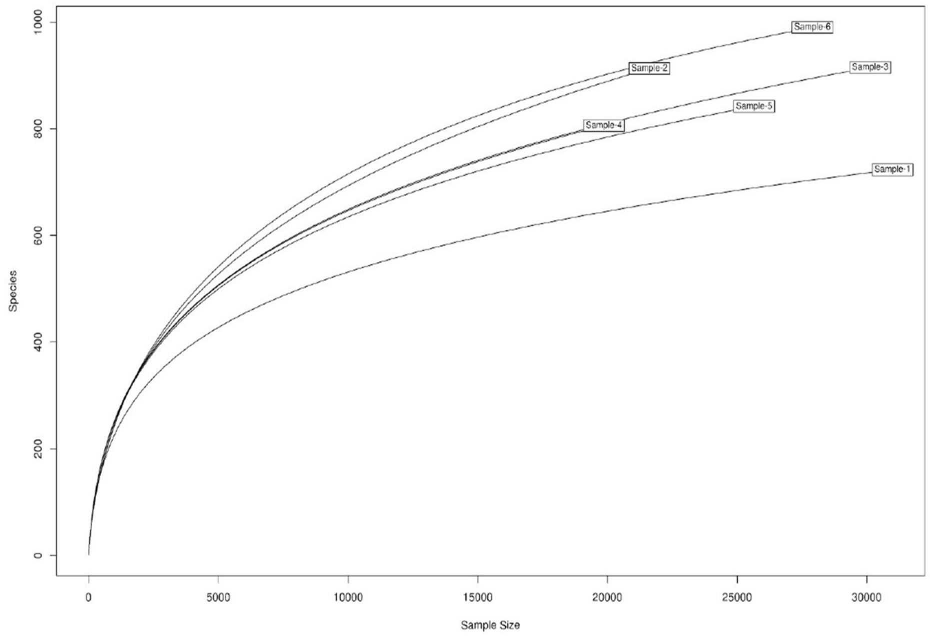

Figure S3(a). Histogram of reads with average sequence quality scores

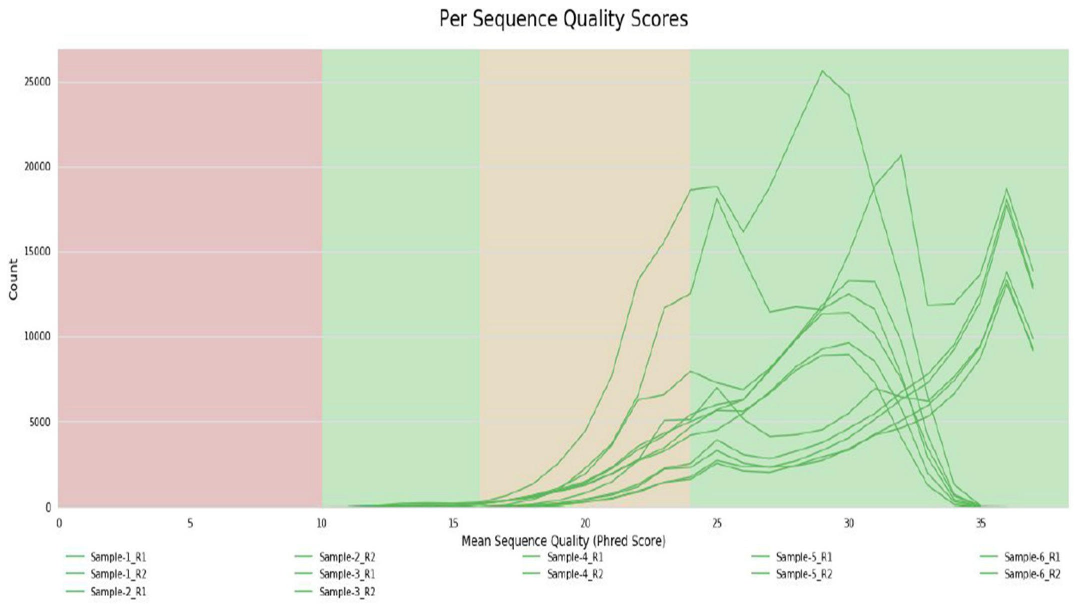

Figure S3(b). Histogram of reads with average base sequence quality scores

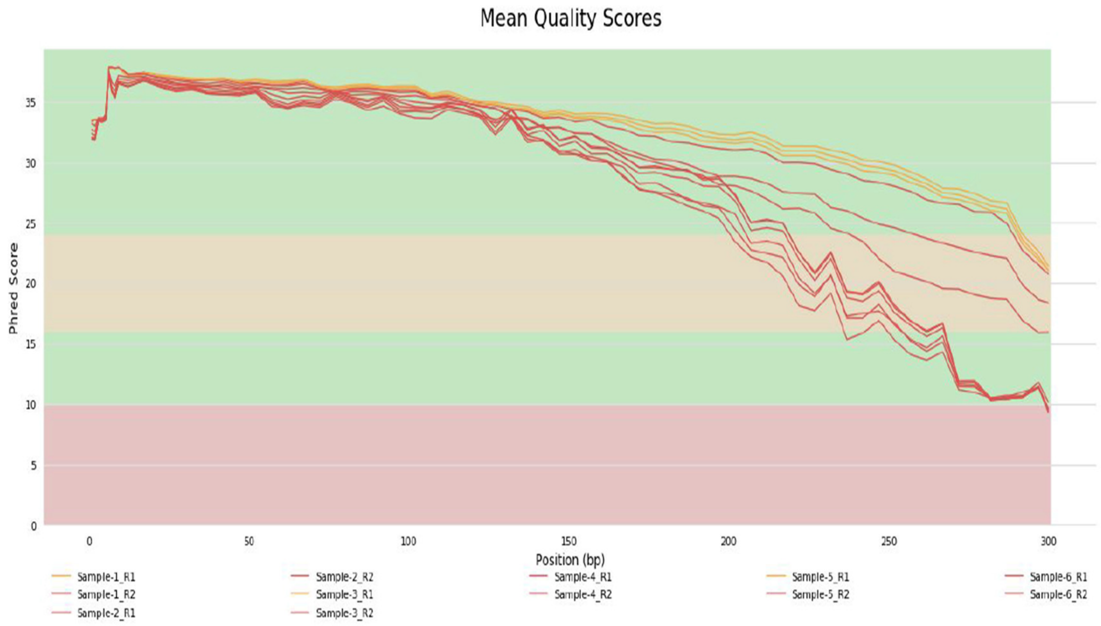

Figure S4 (a-h). Venn Diagram: Selective enrichment of microbial features at arid and semi-arid habitat. The Venn diagrams illustrate analysis of dominant features for arid and semi-arid habitats

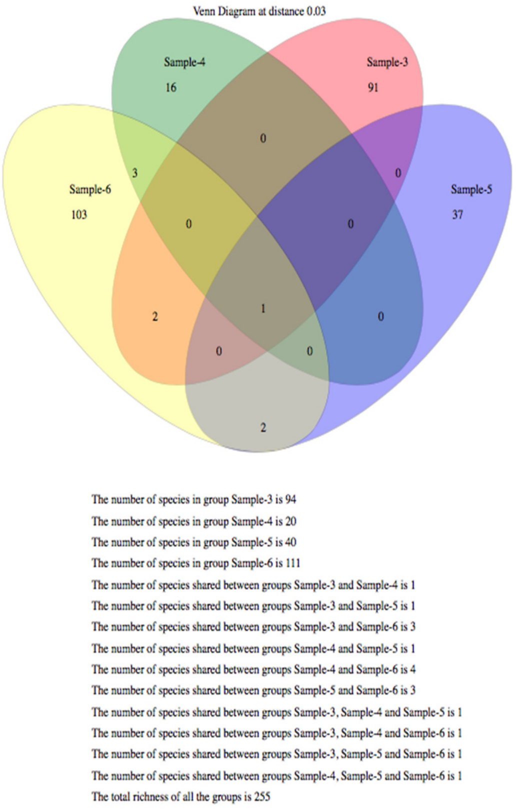
Venn Diagram at distance 0.03
Sample-4
16
Sample-3
91
Sample-6
103
Sample-5
37
The number of species in group Sample-3 is 94
The number of species in group Sample-4 is 20
The number of species in group Sample-5 is 40
The number of species in group Sample-6 is 111
The number of species shared between groups Sample-3 and Sample-4 is 1
The number of species shared between groups Sample-3 and Sample-5 is 1
The number of species shared between groups Sample-3 and Sample-6 is 3
The number of species shared between groups Sample-4 and Sample-5 is 1
The number of species shared between groups Sample-4 and Sample-6 is 4
The number of species shared between groups Sample-5 and Sample-6 is 3
The number of species shared between groups Sample-3, Sample-4 and Sample-5 is 1
The number of species shared between groups Sample-3, Sample-4 and Sample-6 is 1
The number of species shared between groups Sample-3, Sample-5 and Sample-6 is 1
The number of species shared between groups Sample-4, Sample-5 and Sample-6 is 1
The total richness of all the groups is 255

(a)

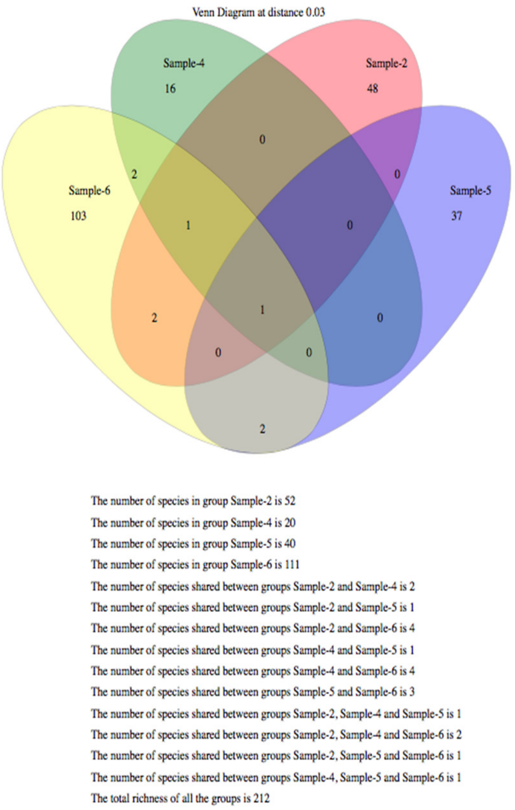
Venn Diagram at distance 0.03
Sample-4
16
Sample-2
48
Sample-6
103
Sample-5
37
The number of species in group Sample-2 is 52
The number of species in group Sample-4 is 20
The number of species in group Sample-5 is 40
The number of species in group Sample-6 is 111
The number of species shared between groups Sample-2 and Sample-4 is 2
The number of species shared between groups Sample-2 and Sample-5 is 1
The number of species shared between groups Sample-2 and Sample-6 is 4
The number of species shared between groups Sample-4 and Sample-5 is 1
The number of species shared between groups Sample-4 and Sample-6 is 4
The number of species shared between groups Sample-5 and Sample-6 is 3
The number of species shared between groups Sample-2, Sample-4 and Sample-5 is 1
The number of species shared between groups Sample-2, Sample-4 and Sample-6 is 2
The number of species shared between groups Sample-2, Sample-5 and Sample-6 is 1
The number of species shared between groups Sample-4, Sample-5 and Sample-6 is 1
The total richness of all the groups is 212

(b)

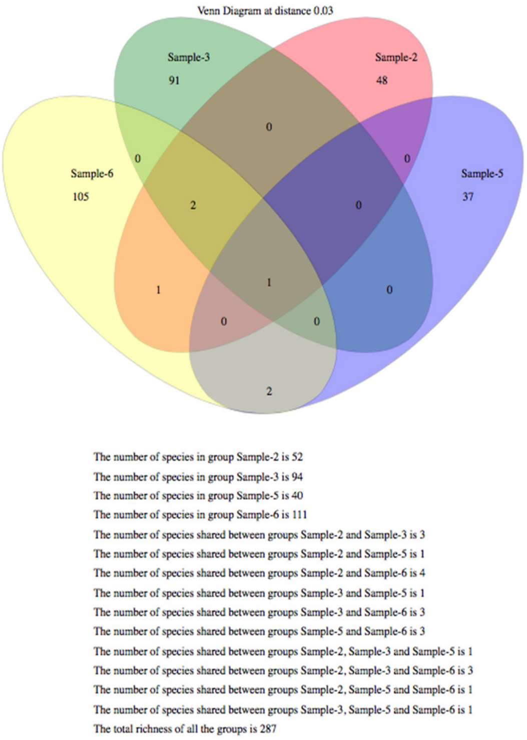
Venn Diagram at distance 0.03
Sample-3
91
Sample-2
48
Sample-6
105
Sample-5
37
The number of species in group Sample-2 is 52
The number of species in group Sample-3 is 94
The number of species in group Sample-5 is 40
The number of species in group Sample-6 is 111
The number of species shared between groups Sample-2 and Sample-3 is 3
The number of species shared between groups Sample-2 and Sample-5 is 1
The number of species shared between groups Sample-2 and Sample-6 is 4
The number of species shared between groups Sample-3 and Sample-5 is 1
The number of species shared between groups Sample-3 and Sample-6 is 3
The number of species shared between groups Sample-5 and Sample-6 is 3
The number of species shared between groups Sample-2, Sample-3 and Sample-5 is 1
The number of species shared between groups Sample-2, Sample-3 and Sample-6 is 3
The number of species shared between groups Sample-2, Sample-5 and Sample-6 is 1
The number of species shared between groups Sample-3, Sample-5 and Sample-6 is 1
The total richness of all the groups is 287

(c)

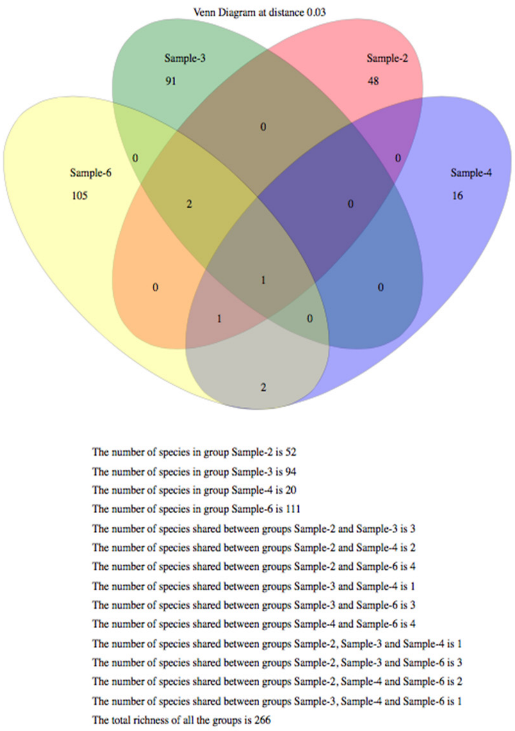
Venn Diagram at distance 0.03
Sample-3
91
Sample-2
48
Sample-6
105
Sample-4
16
The number of species in group Sample-2 is 52
The number of species in group Sample-3 is 94
The number of species in group Sample-4 is 20
The number of species in group Sample-6 is 111
The number of species shared between groups Sample-2 and Sample-3 is 3
The number of species shared between groups Sample-2 and Sample-4 is 2
The number of species shared between groups Sample-2 and Sample-6 is 4
The number of species shared between groups Sample-3 and Sample-4 is 1
The number of species shared between groups Sample-3 and Sample-6 is 3
The number of species shared between groups Sample-4 and Sample-6 is 4
The number of species shared between groups Sample-2, Sample-3 and Sample-4 is 1
The number of species shared between groups Sample-2, Sample-3 and Sample-6 is 3
The number of species shared between groups Sample-2, Sample-4 and Sample-6 is 2
The number of species shared between groups Sample-3, Sample-4 and Sample-6 is 1
The total richness of all the groups is 266

(d)

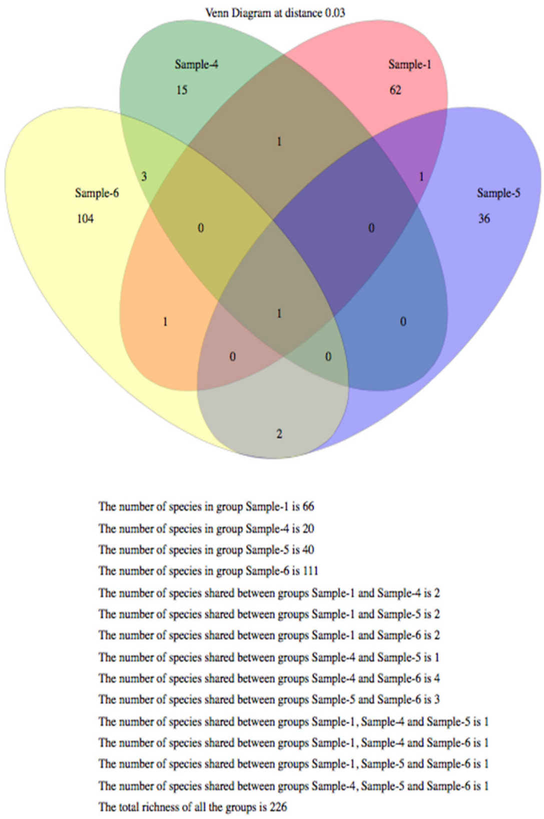
Venn Diagram at distance 0.03
Sample-4
15
Sample-1
62
Sample-6
104
Sample-5
36
The number of species in group Sample-1 is 66
The number of species in group Sample-4 is 20
The number of species in group Sample-5 is 40
The number of species in group Sample-6 is 111
The number of species shared between groups Sample-1 and Sample-4 is 2
The number of species shared between groups Sample-1 and Sample-5 is 2
The number of species shared between groups Sample-1 and Sample-6 is 2
The number of species shared between groups Sample-4 and Sample-5 is 1
The number of species shared between groups Sample-4 and Sample-6 is 4
The number of species shared between groups Sample-5 and Sample-6 is 3
The number of species shared between groups Sample-1, Sample-4 and Sample-5 is 1
The number of species shared between groups Sample-1, Sample-4 and Sample-6 is 1
The number of species shared between groups Sample-1, Sample-5 and Sample-6 is 1
The number of species shared between groups Sample-4, Sample-5 and Sample-6 is 1
The total richness of all the groups is 226

(e)

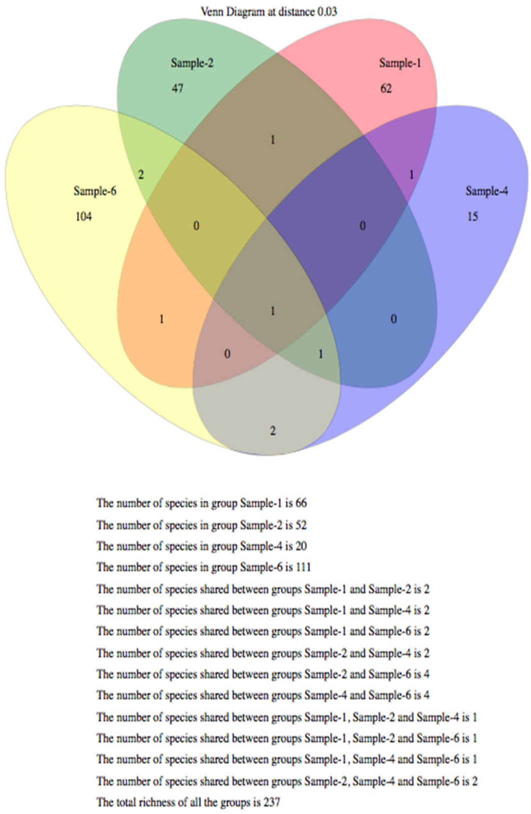
Venn Diagram at distance 0.03
Sample-2
47
Sample-1
62
Sample-6
104
Sample-4
15
The number of species in group Sample-1 is 66
The number of species in group Sample-2 is 52
The number of species in group Sample-4 is 20
The number of species in group Sample-6 is 111
The number of species shared between groups Sample-1 and Sample-2 is 2
The number of species shared between groups Sample-1 and Sample-4 is 2
The number of species shared between groups Sample-1 and Sample-6 is 2
The number of species shared between groups Sample-2 and Sample-4 is 2
The number of species shared between groups Sample-2 and Sample-6 is 4
The number of species shared between groups Sample-4 and Sample-6 is 4
The number of species shared between groups Sample-1, Sample-2 and Sample-4 is 1
The number of species shared between groups Sample-1, Sample-2 and Sample-6 is 1
The number of species shared between groups Sample-1, Sample-4 and Sample-6 is 1
The number of species shared between groups Sample-2, Sample-4 and Sample-6 is 2
The total richness of all the groups is 237

(f)

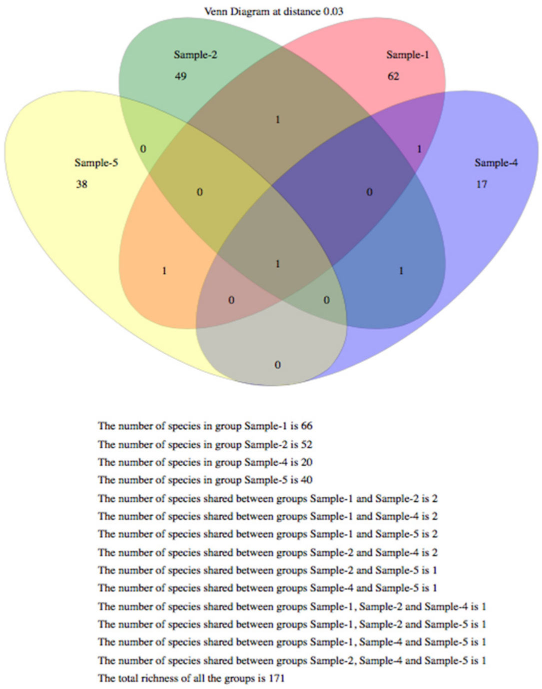
Venn Diagram at distance 0.03
Sample-2
49
Sample-1
62
Sample-5
38
Sample-4
17
The number of species in group Sample-1 is 66
The number of species in group Sample-2 is 52
The number of species in group Sample-4 is 20
The number of species in group Sample-5 is 40
The number of species shared between groups Sample-1 and Sample-2 is 2
The number of species shared between groups Sample-1 and Sample-4 is 2
The number of species shared between groups Sample-1 and Sample-5 is 2
The number of species shared between groups Sample-2 and Sample-4 is 2
The number of species shared between groups Sample-2 and Sample-5 is 1
The number of species shared between groups Sample-4 and Sample-5 is 1
The number of species shared between groups Sample-1, Sample-2 and Sample-4 is 1
The number of species shared between groups Sample-1, Sample-2 and Sample-5 is 1
The number of species shared between groups Sample-1, Sample-4 and Sample-5 is 1
The number of species shared between groups Sample-2, Sample-4 and Sample-5 is 1
The total richness of all the groups is 171

(g)

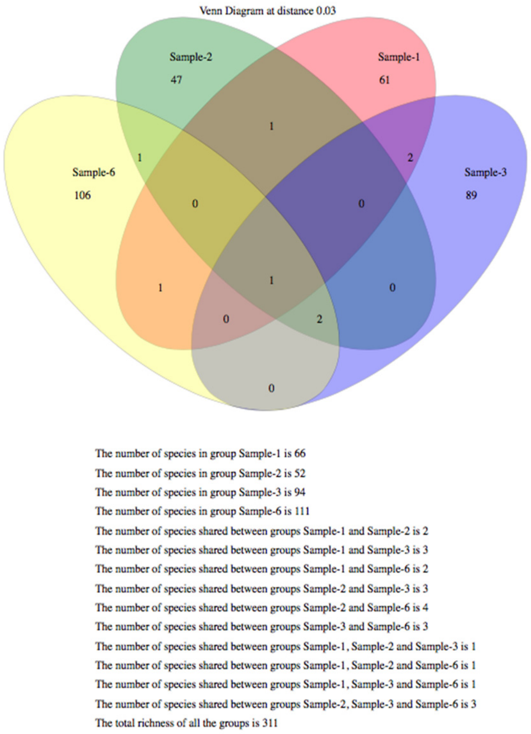
Venn Diagram at distance 0.03
Sample-2
47
Sample-1
61
Sample-6
106
Sample-3
89
The number of species in group Sample-1 is 66
The number of species in group Sample-2 is 52
The number of species in group Sample-3 is 94
The number of species in group Sample-6 is 111
The number of species shared between groups Sample-1 and Sample-2 is 2
The number of species shared between groups Sample-1 and Sample-3 is 3
The number of species shared between groups Sample-1 and Sample-6 is 2
The number of species shared between groups Sample-2 and Sample-3 is 3
The number of species shared between groups Sample-2 and Sample-6 is 4
The number of species shared between groups Sample-3 and Sample-6 is 3
The number of species shared between groups Sample-1, Sample-2 and Sample-3 is 1
The number of species shared between groups Sample-1, Sample-2 and Sample-6 is 1
The number of species shared between groups Sample-1, Sample-3 and Sample-6 is 1
The number of species shared between groups Sample-2, Sample-3 and Sample-6 is 3
The total richness of all the groups is 311

(h)

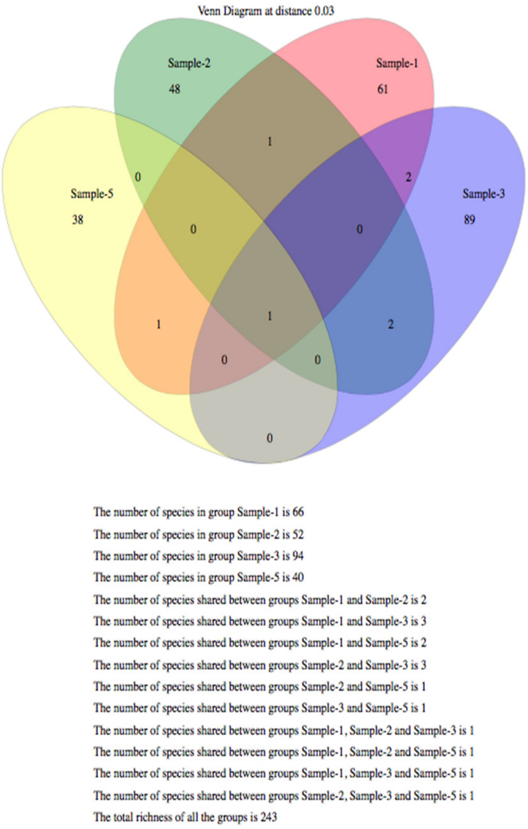
Venn Diagram at distance 0.03
Sample-2
48
Sample-1
61
Sample-5
38
Sample-3
89
The number of species in group Sample-1 is 66
The number of species in group Sample-2 is 52
The number of species in group Sample-3 is 94
The number of species in group Sample-5 is 40
The number of species shared between groups Sample-1 and Sample-2 is 2
The number of species shared between groups Sample-1 and Sample-3 is 3
The number of species shared between groups Sample-1 and Sample-5 is 2
The number of species shared between groups Sample-2 and Sample-3 is 3
The number of species shared between groups Sample-2 and Sample-5 is 1
The number of species shared between groups Sample-3 and Sample-5 is 1
The number of species shared between groups Sample-1, Sample-2 and Sample-3 is 1
The number of species shared between groups Sample-1, Sample-2 and Sample-5 is 1
The number of species shared between groups Sample-1, Sample-3 and Sample-5 is 1
The number of species shared between groups Sample-2, Sample-3 and Sample-5 is 1
The total richness of all the groups is 243

(i)

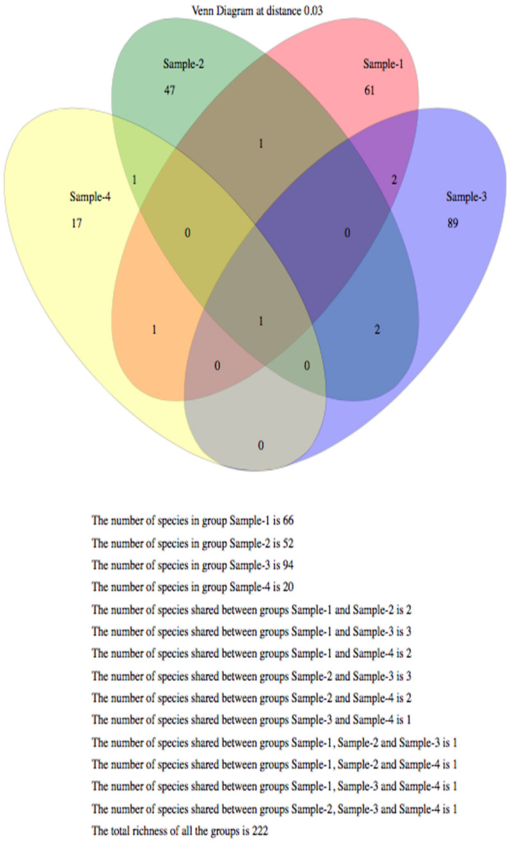
Venn Diagram at distance 0.03
Sample-2
47
Sample-1
61
Sample-4
17
Sample-3
89
The number of species in group Sample-1 is 66
The number of species in group Sample-2 is 52
The number of species in group Sample-3 is 94
The number of species in group Sample-4 is 20
The number of species shared between groups Sample-1 and Sample-2 is 2
The number of species shared between groups Sample-1 and Sample-3 is 3
The number of species shared between groups Sample-1 and Sample-4 is 2
The number of species shared between groups Sample-2 and Sample-3 is 3
The number of species shared between groups Sample-2 and Sample-4 is 2
The number of species shared between groups Sample-3 and Sample-4 is 1
The number of species shared between groups Sample-1, Sample-2 and Sample-3 is 1
The number of species shared between groups Sample-1, Sample-2 and Sample-4 is 1
The number of species shared between groups Sample-1, Sample-3 and Sample-4 is 1
The number of species shared between groups Sample-2, Sample-3 and Sample-4 is 1
The total richness of all the groups is 222

(j)

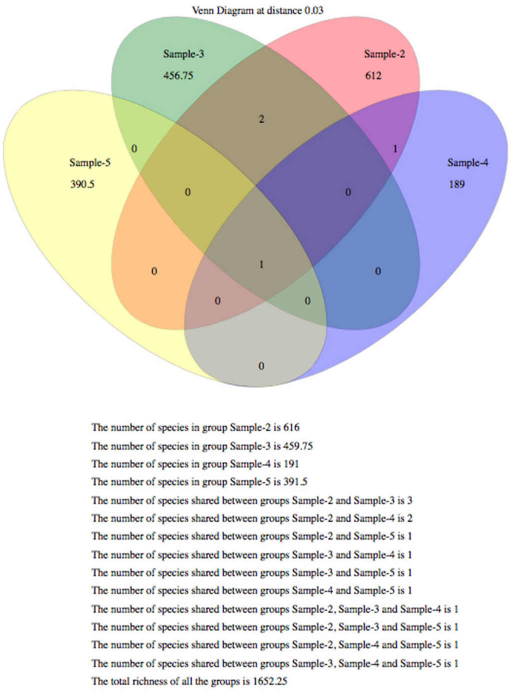
Venn Diagram at distance 0.03
Sample-3
456.75
Sample-2
612
Sample-5
390.5
Sample-4
189
The number of species in group Sample-2 is 616
The number of species in group Sample-3 is 459.75
The number of species in group Sample-4 is 191
The number of species in group Sample-5 is 391.5
The number of species shared between groups Sample-2 and Sample-3 is 3
The number of species shared between groups Sample-2 and Sample-4 is 2
The number of species shared between groups Sample-2 and Sample-5 is 1
The number of species shared between groups Sample-3 and Sample-4 is 1
The number of species shared between groups Sample-3 and Sample-5 is 1
The number of species shared between groups Sample-4 and Sample-5 is 1
The number of species shared between groups Sample-2, Sample-3 and Sample-4 is 1
The number of species shared between groups Sample-2, Sample-3 and Sample-5 is 1
The number of species shared between groups Sample-2, Sample-4 and Sample-5 is 1
The number of species shared between groups Sample-3, Sample-4 and Sample-5 is 1
The total richness of all the groups is 1652.25

(k)

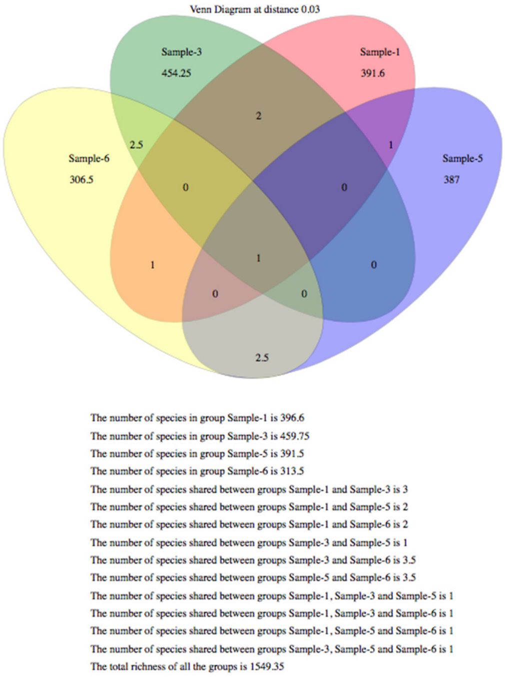
Venn Diagram at distance 0.03
Sample-3
454.25
Sample-1
391.6
Sample-6
306.5
Sample-5
387
The number of species in group Sample-1 is 396.6
The number of species in group Sample-3 is 459.75
The number of species in group Sample-5 is 391.5
The number of species in group Sample-6 is 313.5
The number of species shared between groups Sample-1 and Sample-3 is 3
The number of species shared between groups Sample-1 and Sample-5 is 2
The number of species shared between groups Sample-1 and Sample-6 is 2
The number of species shared between groups Sample-3 and Sample-5 is 1
The number of species shared between groups Sample-3 and Sample-6 is 3.5
The number of species shared between groups Sample-5 and Sample-6 is 3.5
The number of species shared between groups Sample-1, Sample-3 and Sample-5 is 1
The number of species shared between groups Sample-1, Sample-3 and Sample-6 is 1
The number of species shared between groups Sample-1, Sample-5 and Sample-6 is 1
The number of species shared between groups Sample-3, Sample-5 and Sample-6 is 1
The total richness of all the groups is 1549.35

(l)

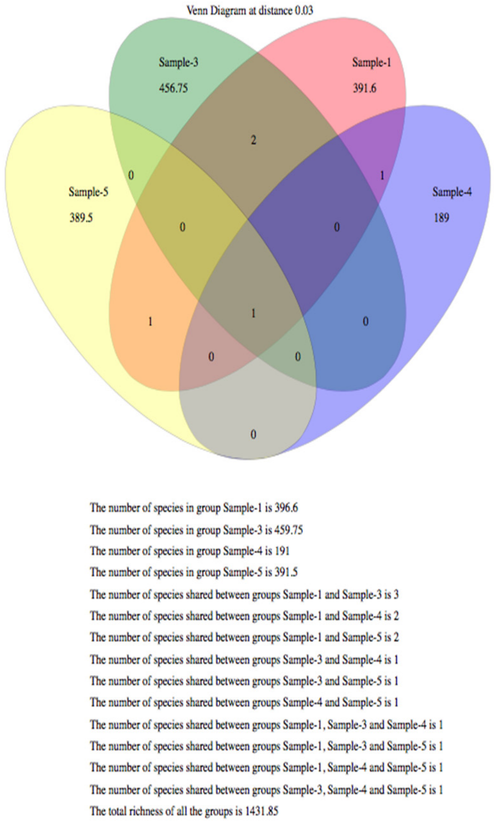
Venn Diagram at distance 0.03
Sample-3
456.75
Sample-1
391.6
Sample-5
389.5
Sample-4
189
2
0
1
0
0
1
1
0
0
0
0
The number of species in group Sample-1 is 396.6
The number of species in group Sample-3 is 459.75
The number of species in group Sample-4 is 191
The number of species in group Sample-5 is 391.5
The number of species shared between groups Sample-1 and Sample-3 is 3
The number of species shared between groups Sample-1 and Sample-4 is 2
The number of species shared between groups Sample-1 and Sample-5 is 2
The number of species shared between groups Sample-3 and Sample-4 is 1
The number of species shared between groups Sample-3 and Sample-5 is 1
The number of species shared between groups Sample-4 and Sample-5 is 1
The number of species shared between groups Sample-1, Sample-3 and Sample-4 is 1
The number of species shared between groups Sample-1, Sample-3 and Sample-5 is 1
The number of species shared between groups Sample-1, Sample-4 and Sample-5 is 1
The number of species shared between groups Sample-3, Sample-4 and Sample-5 is 1
The total richness of all the groups is 1431.85

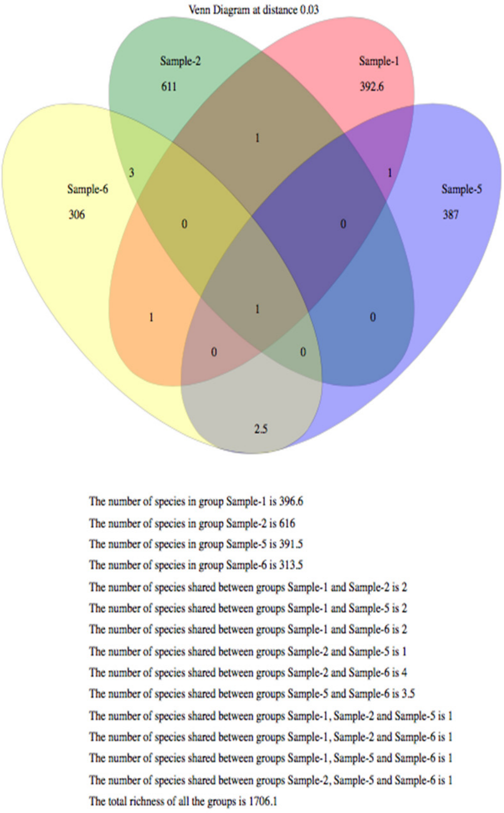
Venn Diagram at distance 0.03
Sample-2
611
Sample-1
392.6
Sample-6
306
Sample-5
387
1
3
1
0
0
1
1
0
0
0
2.5
The number of species in group Sample-1 is 396.6
The number of species in group Sample-2 is 616
The number of species in group Sample-5 is 391.5
The number of species in group Sample-6 is 313.5
The number of species shared between groups Sample-1 and Sample-2 is 2
The number of species shared between groups Sample-1 and Sample-5 is 2
The number of species shared between groups Sample-1 and Sample-6 is 2
The number of species shared between groups Sample-2 and Sample-5 is 1
The number of species shared between groups Sample-2 and Sample-6 is 4
The number of species shared between groups Sample-5 and Sample-6 is 3.5
The number of species shared between groups Sample-1, Sample-2 and Sample-5 is 1
The number of species shared between groups Sample-1, Sample-2 and Sample-6 is 1
The number of species shared between groups Sample-1, Sample-5 and Sample-6 is 1
The number of species shared between groups Sample-2, Sample-5 and Sample-6 is 1
The total richness of all the groups is 1706.1

(m)

(n)

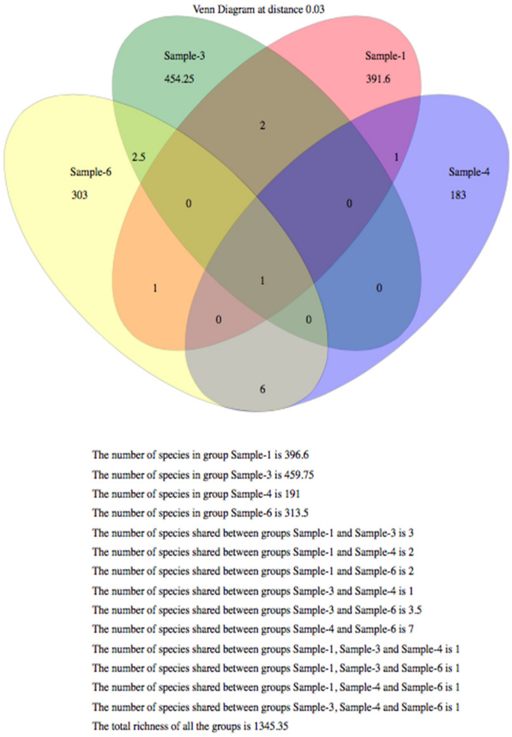
Venn Diagram at distance 0.03
Sample-3
454.25
Sample-1
391.6
Sample-6
303
Sample-4
183
2
2.5
1
0
0
1
1
0
0
0
6
The number of species in group Sample-1 is 396.6
The number of species in group Sample-3 is 459.75
The number of species in group Sample-4 is 191
The number of species in group Sample-6 is 313.5
The number of species shared between groups Sample-1 and Sample-3 is 3
The number of species shared between groups Sample-1 and Sample-4 is 2
The number of species shared between groups Sample-1 and Sample-6 is 2
The number of species shared between groups Sample-3 and Sample-4 is 1
The number of species shared between groups Sample-3 and Sample-6 is 3.5
The number of species shared between groups Sample-4 and Sample-6 is 7
The number of species shared between groups Sample-1, Sample-3 and Sample-4 is 1
The number of species shared between groups Sample-1, Sample-3 and Sample-6 is 1
The number of species shared between groups Sample-1, Sample-4 and Sample-6 is 1
The number of species shared between groups Sample-3, Sample-4 and Sample-6 is 1
The total richness of all the groups is 1345.35

(o)
