# Supplementary material for: Spatial Metagenomic Analysis in Understanding the Microbial Diversity of Thar Desert
Source: Biology (Basel). 2022 Mar 17;11(3):461. doi: 10.3390/biology11030461 (PMC8945486; doi:10.3390/biology11030461)
Supplement: Supplementary file 1 [file biology-11-00461-s001.zip › Supplementary Table 1-6.pdf]

Table S1. Site information and Sample type**.**

**S.I. No.**	**Location**	**Sample count**	**Latitude**	**Longitude**	**Zone**	**Elevation**	**Average Humidity**	**Average Precipitation**	**Average Temperature at sampling**
**1.**	Sihdar, Jaisalmer	6 (soil)	26.2154° N	70.6336° E	Arid	224m	40%	2mm	55°C
**2.**	Lakh granite mine, Jaisalmer	6 (soil)	26.15511 N	70.90740 E	Arid	224m	50%	6mm	53°C
**3.**	Thumbli Coal Plant, Barmer	6 (soil)	26.2032° N	72.4791° E	Arid	207m	53%	2.5mm	56°C
**4.**	Pachpdara lake, Barmer	6 (soil)	25.9351° N	72.2455° E	Semi-Arid	102m	40%	4mm	55°C
**5.**	Khetolai, Jaisalmer	6 (soil)	27.0240714° N	71.7097069° E	Semi-Arid	215m	65%	0mm	55°C
**6.**	Loharki, Jaisalmer	6 (water)	27.1475960° N	71.7649355° E	Semi-Arid	238m	62%	0.5mm	56°C

Table S2. Soil profiling of soil and water samples

Samples	pH	EC (dSm^{-1})	OC (g kg^{-1})	TP (g kg^{-1})	K (g kg^{-1})	Zn (ppm)	Fe (ppm)	Cu (ppm)	Mn (ppm)
**1.**	8.45	0-09	0.16	30	230	0.48	3.84	0.22	2.18
**2.**	8.08	0-81	0.18	32	245	0.52	4.64	0.18	3.24
**3.**	7.72	1-96	0.19	28	182	0.76	5.25	0.16	2.98
**4.**	9.05	13.89	0.26	32	200	0.36	5.24	0.28	2.14
**5.**	8.36	0.23	0.18	40	350	0.46	4.32	0.72	2.34
**6.**	8.22	0.72	7.00 ppm	0.001 mg/ml	245	0.62	0.19	2.32	0.09

Table S3. Library quantity estimate using Qubit HS kit

S. No.	Sample ID	Qubit conc. (ng/ µl)	QC status
**1**	Sample 1	110	passed
**2**	Sample 2	120	passed
**3**	Sample 3	114	passed
**4**	Sample 4	108	passed
**5**	Sample 5	114	passed
**6**	Sample 6	102	Passed

Table S4. Summary of Raw sequence data and quality

Sample-ID	Number of reads	Read Length	GC%	% Bases > Q20
**Sample-1**	185546	301	57.5	98.71
**Sample-2**	210502	301	53.5	98.58
**Sample-3**	203750	301	56	98.78
**Sample-4**	150900	301	57	98.21
**Sample-5**	151590	301	57	98.47
**Sample-6**	459788	301	50	98.51

Table S5. Top 10 OTU's abundance among the samples

Phylum	Class	Order	Family	Genus	1	2	3	4	5	6
**Proteobacteria**	Alphaproteobacteria	-	-	-	558	356	1050	630	697	298
**Planctomycetes**	Planctomycetacia	Gemmatales	Gemmataceae	uncultured	1303	65	155	230	682	341
**Actinobacteria**	Actinobacteria	-	-	-	699	481	647	255	615	55
**Proteobacteria**	Gammaproteobacteria	Betaproteo bacteriales	Burkholderiaceae	Curvibacter	0	0	1	0	0	2378
**Patescibacteria**	Parcubactera	Candidatus_ Kaiserbacteria	Candidatus_ Kaiserbacteria_fa	Candidatus_Kaiserbacteria_ ge	11	17	138	52	15	2136
**Chlamydiae**	Chlamydiae	Chlamydiales	cvE6	cvE6_ge	0	0	2	0	0	2320
**Planctomycetes**	Phycisphaerae	Tepidisphaerales	WD2101_soil_group	WD2101_soil_group_ ge	653	104	639	148	665	45
**Chloroflexi**	Chloroflexia	Chloroflexales	Roseiflexaceae	uncultured	963	28	437	240	460	50
**Actinobacteria**	-	-	-	-	649	275	476	179	489	26
**Proteobacteria**	Alphaproteobacteria	Rhizobiales	Beijerinckiaceae	Microvirga	599	234	591	184	420	32

Table S6. Top 10 Phyla abundance among the samples

Phylum	Sample-1	Sample-2	Sample-3	Sample-4	Sample-5	Sample-6
**Proteobacteria**	5789	4850	7368	3615	4828	8764
**Actinobacteria**	5544	5365	4916	1918	3990	876
**Planctomycetes**	3547	1442	2398	1309	3062	1489
**Chloroflexi**	2735	3064	3088	1034	2441	463
**Bacteroidetes**	1454	1677	813	1509	1021	3260
**Euryarchaeota**	870	139	1107	4836	1097	277
**Firmicutes**	2221	1281	1448	686	771	1844
**Patescibacteria**	162	178	1227	315	323	2896
**Acidobacteria**	895	219	648	296	944	365
**Chlamydiae**	42	17	36	17	43	2453
